# Photovoltaic properties of PSi impregnated with eumelanin

**DOI:** 10.1186/1556-276X-7-377

**Published:** 2012-07-09

**Authors:** Guido Mula, Laura Manca, Susanna Setzu, Alessandro Pezzella

**Affiliations:** 1 Dipartimento di Fisica, Cittadella Universitaria di Monserrato, Università degli Studi di Cagliari, S.P. 8 km 0.7, Monserrato (Ca), 09042, Italy; 2 Dipartimento di Scienze Chimiche, Università degli Studi di Napoli Federico II, Complesso Universitario di Monte Sant'Angelo, Via Cintia, Napoli, 4-80126, Italy

**Keywords:** Organic-inorganic interface, Hybrid material, Bulk heterojunction, Eumelanin, Porous silicon, Photoconversion, 5,6-dihydroxyindole, 88.40.fh, 88.40.jj

## Abstract

A bulk heterojunction of porous silicon and eumelanin, where the columnar pores of porous silicon are filled with eumelanin, is proposed as a new organic-inorganic hybrid material for photovoltaic applications. The addition of eumelanin, whose absorption in the near infrared region is significantly higher than porous silicon, should greatly enhance the light absorption capabilities of the empty porous silicon matrix, which are very low in the low energy side of the visible spectral range (from about 600 nm downwards). The experimental results show that indeed the photocarrier collection efficiency at longer wavelengths in eumelanin-impregnated samples is clearly higher with respect to empty porous silicon matrices.

## Background

The relevance of solar power in the renewable energy field is constantly increasing due to its ready availability and to the fact that the available amount exceeds by several orders of magnitude the needs of the human race. The search for new materials with better performances than standard Si-based solar cells is also constantly increasing. Organic materials [1] emerged as a very attractive solution for this scope, their lower efficiencies with respect to inorganic materials being compensated by lower fabrication costs and higher flexibility. Hybrid materials have also been investigated as a way to combine the low production costs of organic materials with the high efficiency of inorganic materials [2-8].

Among the adopted strategies for new materials, interface geometry often plays a major role in the collection of photogenerated carriers, and bulk heterojunctions [3,9-12] - intimately mixing the two junction materials while keeping them separate in a ‘fractal like’ high-surface interface - are a very promising design for solar cells. This concept was introduced in the mid 1990s for organic solar cells [5,13-15] to shorten the exciton travel distance from the photon absorption site towards the charge-separating interface and then both to reduce the spontaneous recombination and to increase the collection efficiency. The very large interfacial area available for charge separation processes also increases the carrier collection efficiency.

We investigate here a new hybrid material for photovoltaic applications composed by *n*-type porous silicon (PSi) and eumelanin, a natural pigment featuring relatively high electrical conductivity [16] and believed to rely mainly on proton-based conduction [16-18]. Porous Si is a large specific area material, whose properties depend on Si substrate doping and on fabrication parameters [19]. Its application span ranges from biosensor [19] to drug delivery [20] and optoelectronics [21]. In the photovoltaic field, PSi has been considered up to now mainly as an antireflection coating for crystalline Si [22], and there are very few studies about its photovoltaic properties [23,24]. Porous Si-organic hybrids have recently been considered [25], but literature reports are mainly on amorphous [26] or crystalline [27,28] Si. An exception are the interesting results by Nahor et al. [25] reporting a study of a PSi-organic hybrid material realized using conjugate polymers for solar cells, which highlight the potential of PSi-based bulk heterojunctions.

Melanins are a class of natural pigments responsible for the colorations of human skin and hair [29] in the range from light, yellow-reddish (pheomelanins) and dark, brownish black (eumelanins). Their unique status among natural pigments is due to their socioeconomic and biomedical relevance, encompassing racial pigmentation, skin photoprotection, sun tanning, and pigmentary disorders. Moreover, they display a quite unusual set of physicochemical properties such as broadband monotonic absorption in the ultraviolet-visible [18].

These features have suggested the possible use of synthetic (artificial) eumelanins in the development of a new generation of bioinspired electrically active devices [30-32]. More recently, eumelanin biopolymers have also been proposed for optoelectronic and photovoltaic applications [33,34]. Due to their wide absorbance covering the whole visible light spectrum, eumelanins behave as very efficient photoreceptors [31].

As a part of a large project aimed to assess the scope of melanins and melanin-like materials for the development of novel hybrid functional architectures [35,36], we investigate here ‘5,6-dihydroxyindole-melanin immobilized PSi’ as a prototypal device for the generation of white light-induced photocurrent.

## Methods

### Porous silicon

**Figure 1:**
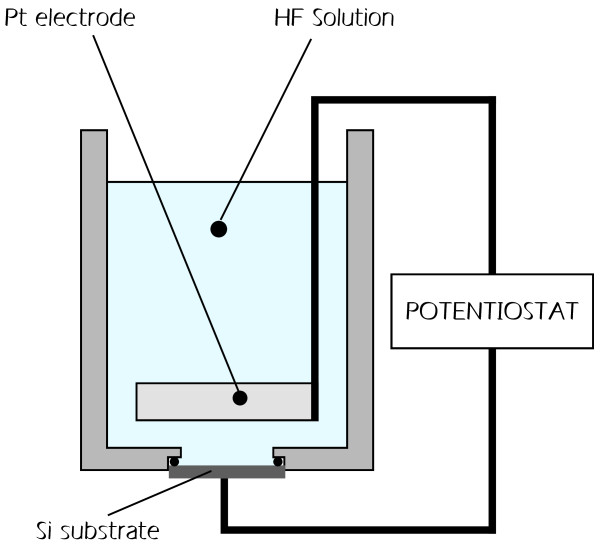
**Schematic of PSi fabrication equipment.** The working electrode is the crystalline Si wafer.

PSi samples were prepared by electrochemical etch in the dark of (100)-oriented, phosphorous doped, *n*^+^-type monocrystalline Si wafers from Siltronix (Archamps Technopole, Archamps, France) using the procedure described in [37]. The resistivity of the wafers is in the 0.007 to 0.003 ohm/cm range. The constant current etching process [38,39] has been performed in a polyvinylchloride electrochemical cell using HF:H_2_O:ethanol solution in 15:15:70 percent, respectively. The potential source was a PARSTAT 2273 potentiostat from Princeton Applied Research (Oak Ridge, TN, USA). The various components of the fabrication cell are schematically shown in Figure 1. The silicon substrate acts as the working electrode and a platinum wire or grid is used as a counter electrode.

**Figure 2:**
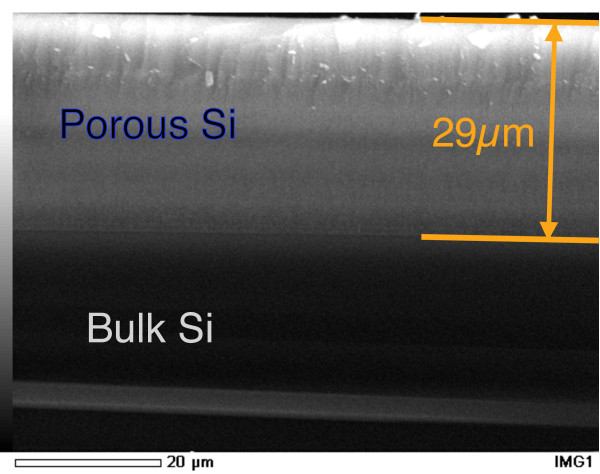
**Cross-section SEM image of a PSi sample for thickness measurement.** The porous layer and the underlying bulk Si are indicated. The PSi external surface and the interface with the bulk Si substrate are indicated with the upper and lower orange lines, respectively. The measured thickness value is also shown.

All samples considered have a nominal 5 μm thickness. The relation between the samples' thickness and their respective fabrication time has been studied by means of scanning electron microscopy (SEM). In Figure 2 we show an example of thickness measurement performed on a 29 μm-thick PSi sample. The scale is shown at the bottom of the image. The porous layer (comprised between the two orange lines) and the bulk Si substrate are indicated, together with the measured thickness value. The thickness measurements proved to be fully reproducible for any thickness below or equal to 29 μm (the thickest sample tested) and directly proportional to the formation time. The samples' porosity, measured by gravimetry, was 55 % (empty/full ratio) for all samples. A few samples have been electrochemically oxidized, in the same cell used for the formation process, using a 0.1 M aqueous solution of KNO_3_ and a constant current *I* = −5 mA. The optical reflectivity measurements were performed using a PerkinElmer (Perkin Elmer Italia SpA, Monza (MI), Italy) Lambda 950 spectrometer equipped with the Universal Reflectance Accessory using an incidence angle of 8°.

### Eumelanin

**Figure 3:**
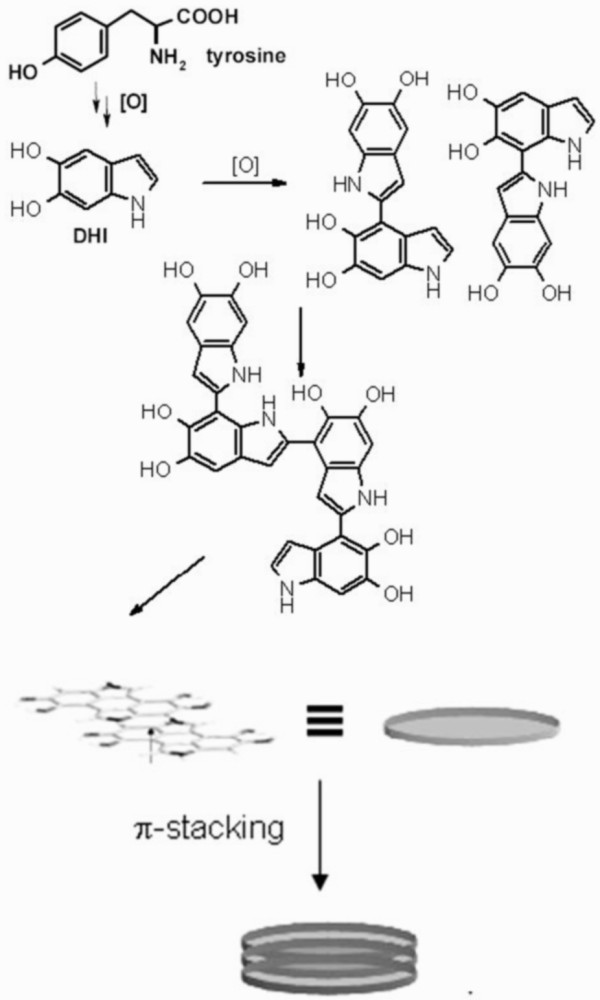
**Schematic path of eumelanin formation **[29]** and proposed hierarchical structure of the pigment.** Early DHI oligomers are shown [40].

Eumelanin formation starts from a tyrosinase-catalyzed oxidation of tyrosine (Figure 3) in a multistep process up to 5,6-dihydroxyindole (DHI) and 5,6 dihydroxyindole-2-carboxylic acid. Oxidative polymerization of these indoles then gives rise to the black-brown variety of melanin biopolymer: the eumelanin [29].

In our experiments, DHI [41] and synthetic eumelanin [42] were prepared according to the literature: a solution of DHI (50 mmol) in phosphate buffer, 0.1 M, pH 7.4, (10 ml) was treated with tyrosinase (400 units) under a stream of oxygen for 4 h at 25 °C, then acidified to pH 4.5 and washed first with distilled water and then in methanol/water 7/3.

Eumelanin immobilization on porous Si was obtained by treating the substrates with the appropriate solution/suspension of DHI or synthetic melanin: methanol, methanol/water, or methanol/phosphate buffer pH 7.0 were used as liquid phase. The substrates were processed with three cycles, 5 min each, in ultrasound bath, followed by 4 h oxygen exposition. When suitable, tyrosinase was also added to the mixture to promote oxidation process.

### Photocurrent measuring procedure

Electrical contacts were realized by deposition of gold spots on top of the empty and impregnated porous layers by sputtering using an Emitech K450 sputter coater (Quorum Technologies Ltd, East Grinstead, West Sussex, UK). On each sample, four contacts were realized to test the reproducibility of the procedure.

The samples' photoconductivity was tested using a PM8 Analytical prober and a Keithley multimeter (Keithley Instruments Inc., Cleveland, OH, USA). The light source was a tungsten-halogen lamp, whose spectral range at the output of the optical system was in the 400 to 850 nm interval. The active external surface of the samples involved in the photocurrent generation is estimated of the order of a squared millimeter.

## Results and discussion

Different approaches for the immobilization of eumelanin in the porous matrix have been explored, including treatment of the porous Si with preformed synthetic eumelanin and *in situ*-induced oxidative polymerization of DHI.

To gain insight into the mode of coupling (Figure 3) of DHI during polymer buildup in this procedure, which is important to match the basic structural features of the immobilized DHI-derived polymer [30,32] and to validate the procedure, mother liquors were collected and the reaction was stopped in the early stages by the addition of sodium dithionite to reduce the oxidized species. The crude oligomer-containing mixture was acetylated according to an established protocol [40] and examined by thin layer chromatography (TLC). This treatment of the mixture allowed the isolation of two main eumelanin oligomer intermediates, which were identified as the acetylated 2,7′- and 2,4′-biindolyls.

The oligomer identification has been performed using the protocols reported in [40] and [43]. In detail, after 2 min of the substrate treatment, the liquors were removed and the oxidation was halted by the addition of 5 % *w/w* aqueous solution of sodium dithionite and acidified to pH 4 with 3 M HCl. The reaction mixture was extracted repeatedly with ethyl acetate (3 × 250 mL), and the combined organic layers were dried over sodium sulfate and taken to dryness.

The residue was acetylated with acetic anhydride-pyridine 95:5 (*v/v*) and fractionated by preparative TLC (CHCl_3_/MeOH 98:2) to identify the constituents by comparison with synthetic standards with known structure.

**Figure 4:**
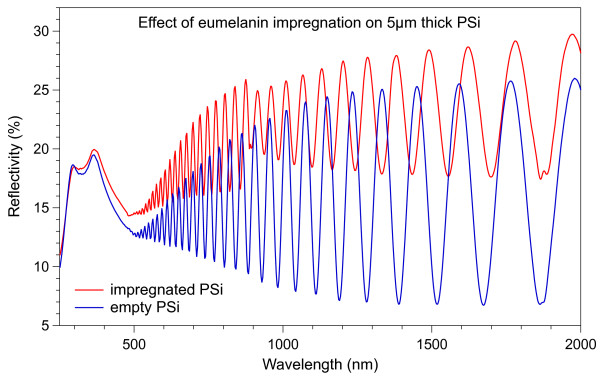
**Absolute optical reflectivity of PSi samples.** The blue curve refers to an empty sample and the red curve to a eumelanin-impregnated PSi layer.

The optical reflectivity measurements were performed on both empty and impregnated PSi matrices to explore the impregnation procedure. In Figure 4 we show optical reflectivity spectra obtained on two PSi samples, where the thin-layer Fabry-Pérot interference fringes are clearly visible. The red curve corresponds to an impregnated PSi sample and the blue curve to an empty one with equal thickness. The discontinuity for wavelengths longer than 890 nm in the red curve is due to instrumental adjustment routine at the detector change. There are two main differences between the two curves. The first one is in the intensity of the interference fringes. The fact that the impregnated sample shows less intense fringes can be attributed to an increase of the absorption coefficient of the layer [44]. The second difference between the two curves is the higher number of fringes per wavelength range for the impregnated sample. This increase can be correlated to an increase of the optical thickness of the sample [44]. Given that the two samples have the same nominal thickness, this difference is clearly related mainly to the change in the refractive index.

**Figure 5:**
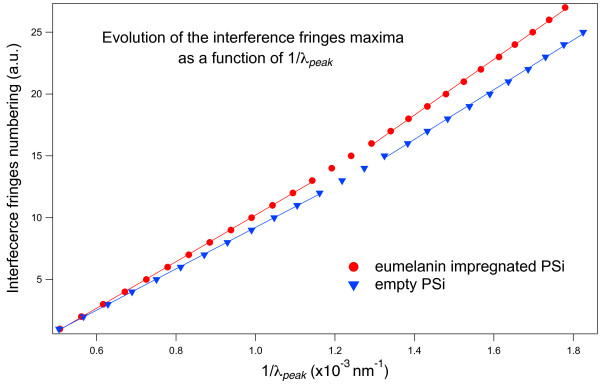
**Interference peak ordinal vs. the inverse of the peak wavelength obtained from the data of Figure**4**.** Blue triangles refer to the empty PSi layer and red circles to the eumelanin-impregnated PSi layer. The linear fits of the experimental data are also shown for the lower (right) and higher (left) energy sides of the explored spectral range.

In Figure 5 we show the dependence of the interference peak maxima number on the inverse of the wavelength for the two samples of Figure 4, the color code being also the same. If the refractive index had been constant, this plot would have been a straight line [45]. In our case, however, considering the large spectral range explored, the refractive index is not constant. For this reason, the curves have been fitted separately for the higher (left) and lower (right) energy sides of the spectra. Since the slopes of the linear fits related to the impregnated sample are always higher than those related to the empty sample, the results of Figure 4 demonstrate that the refractive index of the impregnated PSi layer is higher than that of the empty PSi layer in the whole spectral range examined. From the results of Figures 4 and 5, we can then conclude that the eumelanin impregnation process leads to an increase of both the absorption coefficient and the refractive index of the PSi matrix. Moreover, the absence of beats in the interference fringes of the impregnated layer indicates that the impregnation process is quite homogeneous throughout the whole PSi thickness and that there is no double layer given by a partial, depth-limited, eumelanin pore penetration.

**Figure 6:**
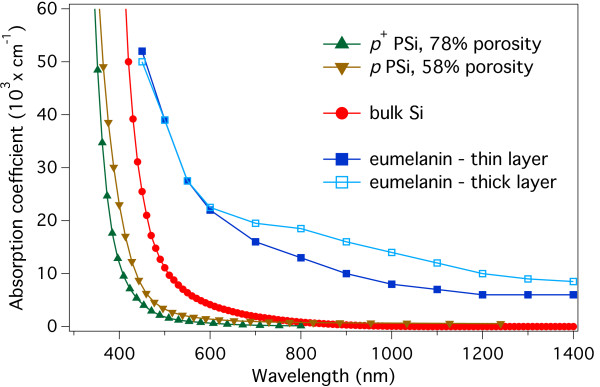
**Literature absorption coefficients dispersion curves for PSi, Si and eumelanin.** Comparison of the absorption coefficients dispersion curves reported in literature for several materials: eumelanin film, thin (80 nm, full squares), and thick (800 nm, empty squares), [16]; 58 % porosity *p*-type (inverted triangles) and 78 % *p*^+^-type (upright triangles) porous Si, [44]; and bulk Si (full circles) [46]. The plot shows how the absorption coefficient of eumelanin is significantly larger than that of PSi, becoming more than one order of magnitude larger for wavelengths longer than about 650 nm. It is worth noting that this is also valid with respect to the undoped bulk Si.

These results are in agreement with the reported literature absorption coefficients of eumelanin and PSi. To show this, in Figure 6, we plot the absorption coefficients of 78 % *p*^+^-type and 58 % *p*-type porous Si [44], bulk Si [46], and eumelanin [16]. The absorption coefficients dispersion curves for eumelanin have been obtained from thin (80 nm) and thick (800 nm) film. All symbols are explained in the figure caption.

The data shown in Figure 6 clearly evidenced that the optical absorption coefficient of both thin and thick eumelanin films is significantly greater than that of PSi and even bulk silicon for photon energies lower than 2.5 eV (500 nm). For wavelengths longer than 600 to 650 nm, the eumelanin absorption coefficient is still significant (well above than 10^4^ cm^−1^) and more than a factor of ten larger than that of PSi whose absolute value becomes less than 10^3^ cm^−1^. This is where the effect of eumelanin may be expected to generate a more significant difference in the photoconductive behavior of empty and impregnated porous layers. It is important to note that from [16], the eumelanin absorption coefficient at 1,400 nm is still more than 6,000 cm^−1^ for thin films and 8,000 cm^−1^ for thick films.

The photoconductive properties of porous Si samples were studied with and without eumelanin for unoxidized layers. A few samples were oxidized at several oxidation levels. This test has been done because, although the partial oxidation process may reduce the PSi conductivity, the oxidation-induced modification of the eumelanin adhesion to the pore walls could, in principle, more than counterbalance the effect. In all cases, however, all the partially oxidized samples showed no measurable photosensitivity. Whatever the impinging light intensity value (up to a maximum of about 200 W/m^2^) is, with and without eumelanin, our results conclusively show that even a thin oxide layer on the pore's wall is sufficient to severely limit the layers' photosensitivity.

All non-oxidized samples, for all eumelanin immobilization approaches considered here, showed a marked photosensitivity. When illuminating with the whole lamp spectrum, the variation in the photocurrent intensity from very low ambient light to the maximum impinging light intensity showed an increase of more than three orders of magnitude, fully reproducible for the same gold plot. However, while the wavelength dependence was highly reproducible, as discussed later in more detail, a variability was observed in the maximum measured photocurrent when using different gold contacts on the same sample and/or on different samples, even if nominally identical. At the same time, the maximum photogenerated voltage showed a much lower variation, being between 100 and 150 mV for impregnated samples and between 120 and 200 mV for empty samples, suggesting a contact-related and non material-related issue. Empty PSi layers showed more stable results with respect to the impregnated samples. The maximum absolute photocurrent values measured with full illumination for impregnated and empty PSi were within about a factor of two, while the photovoltage measurement were much closer (5.1 mA/200 mV for empty PSi and 2.2 mA/150 mV for impregnated samples). The observed variability did not appear to depend on the eumelanin immobilization procedure.

**Figure 7:**
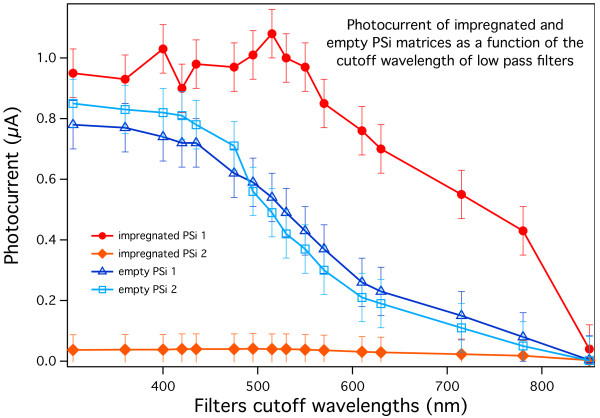
**Photocurrent for four typical porous Si samples.** Two of the samples (full rounds and diamonds) are impregnated with eumelanin and two (empty triangles and squares) are as prepared. The sample labeled ‘impregnated PSi 2’ shows very low photocurrent intensity due to the unoptimized contact fabrication process. The wavelength reported in the abscissa is the cutoff wavelength of the low-pass filters we used.

To better evaluate the effect of the eumelanin insertion on the photoconductive properties of the samples and to discriminate possible contact issues from the samples' behavior, we measured the wavelength dependence of the light response of impregnated and empty porous Si samples using low-pass optical filters in front of the samples. By this approach, using a given filter the light arriving onto the samples' surface will only have wavelengths longer than the cutoff filter's. This means that the photocurrent measured with each filter will be given by all photocarriers generated by the interaction of the samples with the residual spectral range, that is, the part whose photon energy is lower than that characteristic of the cutoff filter. Consequently, if the carrier photogeneration occurs in the whole source spectrum, the plot of the photocurrent as a function of the cutoff wavelengths is expected to be a monotone curve which is increasing when moving towards shorter wavelengths. The results obtained on typical samples in our experiments are shown in Figure 7. The samples indicated as ‘empty PSi 1’ and ‘impregnated PSi 1’ are the same samples whose optical reflectivity spectra have been shown in Figure 4. The absolute current values are comparable (*I*_max_ = 0.8 μA for the two empty PSi layers and *I*_max_ = 0.9 μA for the impregnated sample labeled impregnated PSi 1) for three of the samples shown, while for one of the impregnated samples, the maximum absolute photocurrent is significantly lower (*I*_max_ = 0.09 μA, *V*_max_ = 100 mV with full spectral range illumination).

**Figure 8:**
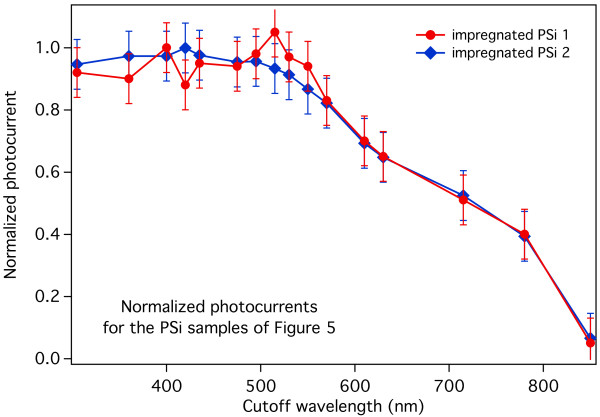
**Normalized photocurrents for the two impregnated PSi samples shown in Figure **7**.** It is worth noting the almost complete superposition of the two behaviors even if the absolute photocurrent values are significantly different.

To separate the contact-related issues from the photogeneration behavior of the samples, the photocurrent intensity and the wavelength dependent photogeneration behavior must be separated. If the behavior of the samples would prove to be the same with respect to the maximum recorded photocurrent, it would be possible to attribute the intensity differences from sample to sample essentially to contact-related issues and not to the hybrid material building procedure adopted. Accordingly, we show in Figure 8 the photocurrents of the two impregnated PSi samples of Figure 7, normalized by dividing each value by the maximum measured photocurrent of that sample. As in Figure 7, the normalized photocurrents are plotted vs. the low-pass filters cutoff wavelengths. Please note, however, that the peak at 500 nm of the impregnated PSi 1 sample, being clearly due to signal noise, has not been used as a reference for the normalization.

The striking feature of Figure 8 is the almost complete superposition of the two curves, indicating that the relative behavior of the two impregnated samples is the same, despite the factor of 20 within their maximum values. This clearly indicates, as stated above, that the observed photocurrent intensity fluctuation is fundamentally related to the contact fabrication process and does not point to a poor reproducibility of the hybrid material behavior.

From the results shown in Figures 7 and 8, there are several considerations that can be made. First, all curves show a monotonic photocurrent increase when going from longer to shorter wavelengths, indicating that the absorption takes place in the whole available light spectrum for both impregnated and empty PSi layers. The second significant feature is that impregnated samples show a steep photocurrent increase in the lower energy part of the spectrum. This is coherent with what can be expected by the comparison of the optical absorption coefficients of the materials, as discussed earlier in this work. If we compare empty and impregnated samples with similar maximum photocurrent, about 40 % of the total photocurrent is generated, for impregnated samples, when using wavelengths longer than 780 nm; while for empty PSi layers, the same wavelength range gives only about 15 % of the maximum photocurrent.

The latter effect is even more significant if we keep in mind the very low intensity of the impinging light in the 700 to 850 nm range: for the impregnated samples, 40 % of the total photocurrent is obtained with a very limited part of the total spectrum; while to obtain the same amount of photocurrent with the empty PSi samples, we need to increase the wavelength range up to 550 nm, including most of the available lamp spectral range.

These results show how the impregnation of the PSi matrix with eumelanin significantly increases the capability of the layer to efficiently photogenerate carriers from light especially when approaching the infrared region.

## Conclusions

We have shown that the photovoltaic properties of PSi may be significantly improved by impregnation with eumelanin. In particular, we showed that introducing the pigment in the porous Si matrix leads to a significantly more efficient photocurrent generation in the lower energy part of the experimental wavelength range explored with respect to empty porous Si layers.

This result not only contributes to expand the scope of heterojunctions in developing a new hybrid material but also provides the first evidence of the eumelanin capability to efficiently collect the photon energy. In a given substrate, eumelanin seems to act as a kind of ‘antenna’, modifying the range of the useful wavelength range for photoconversion application.

Although further experimental and theoretical studies are needed to improve the electrical contacts reproducibility and to reach a deeper understanding of the observed behavior in view of future development and applications, the proof of principle device presented here opens a new window into the evolving panorama of eumelanin-based devices and contributes to the development of bioinspired and biocompatible optoelectronic devices.

## Competing interests

The authors declare that they have no competing interests.

## Authors’ contributions

GM conceived of the study, participated in its design and coordination, prepared part of the samples and performed the reflectivity measurements and analysis. LM performed the photoconductivity measurements and prepared part of the samples. SS participated in the design of the study. AP participated in the design of the study, carried out the eumelanin preparation and performed the PSi impregnation process with eumelanin. All authors contributed to the data analysis. All authors read and approved the final manuscript.
